# Don’t put a tag on us: disability is not a side issue

**DOI:** 10.2471/BLT.16.030216

**Published:** 2016-02-01

## Abstract

Improving the health and well-being of people with disabilities is fraught with challenges. W Aubrey Webson tells Fiona Fleck about the progress made over the last two decades.

Q: What were the formative experiences as you grew up in the Caribbean that shaped your outlook on life and your work on disability in Antigua and Barbuda and other countries?

A: My life circumstances were shaped by my family, rural folk who were never ashamed of me and of the fact that I am blind. There were not many young blind people where we lived. People told my parents I would not survive or that, if I did, I would be a burden on my family. The love of my parents and siblings was crucial for my personal development. They did not try to limit me but allowed me to do all kinds of things other kids did, such as being involved in family life and chores. I was lucky to have an education and to be integrated into social life in Trinidad where I grew up. People with disabilities face many barriers, both physical and attitudinal. I always had people who believed in me and who encouraged me to break down these barriers by allowing me to participate in social and family life.

Q: It is more than 20 years since the General Assembly of the United Nations adopted the Standard Rules on the Equalization of Opportunities for Persons with Disabilities in 1993. What progress has been made since then?

A: In the community in the Caribbean where I grew up, many people lost their sight – including myself – when they were children because of the lack of awareness of the conditions that brought on blindness and a lack of early detection of conditions, such as measles, vitamin A deficiency, *ophthalmia neonatorum* and malaria, by the health services. What the United Nations did was to create a platform to create awareness, building on the international year and international decade for persons with disabilities, and it promotes the use of the rules in the development of national policies related to disability and rehabilitation. Since 1993 there has been a greater awareness of people with disabilities and the challenges for them, and we have seen significant changes. Today there is easier access and information for people who are blind in some countries. For example, in Antigua there is easier physical access for people with disabilities to health clinics than when I was growing up. With today’s improved access to information, we can make better decisions about our health care and family life.

“Since 1993 there has been a greater awareness of people with disabilities and the challenges for them.”

Q: What are the challenges for countries to respond to the needs of people with disabilities and how have you been able to help in your work with NGOs and international organizations?

A: The big challenge in most countries is implementation. A lot of countries have good laws on disability, they have followed the patterns of the international community by developing strong affirmative action laws around disability, such as national policies on education and access. But making this the reality on the ground is difficult and, in addition, it is expensive to implement such laws in developing countries. Disability has often been seen as an area that needs separate structures and a separate budget, thus it is often placed in a silo, and NGOs are left to take responsibilities for this silo. For example, if your child is deaf–blind, that child is shunted from one government institution to another. In many countries disability services are part of the health services, no matter what type of disability. In my own country, the education authorities used to be responsible for disability issues. In other countries the welfare authorities are responsible for disability issues. So NGOs work to make services for the disabled more accessible while building awareness and respect for people with disabilities.

Q: In which developing countries have such efforts been successful?

A: Sometimes things look rosy when built around a champion, but when that champion is removed, the service takes a dive in terms of quality because there is no structure in place. You often see this in the NGO world. In addition, it’s a big economic challenge for developing countries to meet special requirements for assistive devices and technologies that people with disabilities can use to increase their level of independence in their daily life. Some persons with disabilities need equipment to make them equally functioning. For example, getting people who are blind set up and working with a laptop can be more expensive than for people without visual impairments. Over the last 10 years, Uganda and South Africa have passed very good disability friendly laws and political will is strong. But if you asked people with disabilities from those two countries, they would say: “Are we talking about the same country?” In some African countries and in India, for example, there are some really good programmes but implementation is a challenge.

Q: What about your own country?

A: Antigua has done what no other country has done by appointing a person with disabilities to represent it at the United Nations. On other levels, people with disabilities in our country are still struggling.

Q: Disability is referred to in five of the 17 new Sustainable Development Goals (SDGs) of the United Nations. Are disabilities issues addressed adequately in the new SDGs?

A: If you had asked me this question two years ago, my answer would have been very different. Now that I am engaged in the process, I understand better both the NGO and the government perspective. One of the things I and other disability campaigners strived for at the United Nations was to stop disability from being treated as a side issue. I hated the idea that we should “deal with women and girls, and indigenous people and persons with disabilities”, as if women and girls or indigenous people don’t have disabilities. Disability is not a separate issue with a tag on it. I and other campaigners in the disability community worked hard for disability to be given much more attention in the SDGs than was the case in the Millennium Development Goals and we have succeeded. So that is a major step. Now we must push for further progress, by ensuring that disability is measured and monitored.

Q: How do you see the role of WHO with regard to disability?

A: There is a major need for monitoring services to gather data on how many people have disabilities in different countries. In Latin America and the Caribbean, we don’t have enough of these data especially on children, but we need these data to design public health programmes both that address their disabilities and help prevent many of these types of disabilities in future. WHO can certainly help in raising awareness about the role of noncommunicable diseases (NCDs) as many people do not realize that NCDs are a major factor for many disabilities.

Q: For example?

A: Many people with diabetes risk amputations and blindness and more and more people are getting diabetes, especially in low- and middle-income countries, and yet it can be prevented by having a healthy diet with plenty of physical activity and keeping a healthy body weight. Smoking too is a risk factor for diabetes and can also lead to respiratory illness, when an individual’s breathing and movement are severely impaired. We need to detect deafness and hearing loss in children much earlier. Neonatal and infant hearing screening programmes are needed to detect hearing loss early enough to provide effective treatment or management of these conditions with hearing aids. Most cases of blindness can be prevented. For example, vitamin A deficiency is an important cause of preventable blindness in children, while most cases of blindness from glaucoma can be prevented through early detection and treatment.

Q: You have worked to promote the rights and well-being of people with disabilities in many countries, including in Belize, Saint Lucia and South Africa. How did you help those countries?

A: A lot of my work was in education or social development for people with disabilities. I have done a lot of training in self-awareness, helping people to break down the barriers so that they can build self-confidence. For example, in South Africa I was helping to build NGOs for people with disabilities. I was engaged in policy development, improving access and educational rights. 

Q: You helped to formulate policies on different aspects of disability. Can you give examples of these projects?

A: I really enjoyed working on World Bank policy on inclusive education and on employment equity for the Inter-American Development Bank for people with disabilities. The idea was that these policies would be used at national level by implementing them in countries. Championing greater opportunities for persons with disability meant that I could help to break down barriers to their personal development by providing them with education and employment opportunities. That was a thrilling period of my life because I could shape the agenda and how things are done in countries. Persons with disabilities should not have to work harder than others to make their contribution, but very often we have to because there are so many cultural and attitudinal barriers to break down. A key way to break these down is to work alongside people without disabilities. That’s why, for example, employment law and policies that allow for the inclusion of people with disabilities in the workplace are so important.

“Persons with disabilities should not have to work harder than others to make their contribution.”

Q: How will you continue your work on disability as UN Ambassador?

A: I will continue to be an advocate for persons with disabilities, but in a different way: by speaking in the halls of the United Nations. My presence here at the United Nations as a person with disability is the best message you can send. I will continue to address policy issues affecting persons with disabilities, for example in the SDGs. Often people with disabilities are portrayed in the media as heroes or victims and this contributes to the side-issue effect. I refuse to be siloed as a “disability ambassador” and, as already mentioned, I am working to ensure that disability is no longer regarded as a side issue. Disability issues must be incorporated in every issue in the same way gender issues are.

